# Supplementary material for: PPM1H phosphatase counteracts LRRK2 signaling by selectively dephosphorylating Rab proteins
Source: eLife. 2019 Oct 30;8:e50416. doi: 10.7554/eLife.50416 (PMC6850886; doi:10.7554/eLife.50416)
Supplement: Transparent reporting form [file elife-50416-transrepform.docx]

***eLife’s* transparent reporting form**

We encourage authors to provide detailed information *within their submission* to facilitate the interpretation and replication of experiments. Authors can upload supporting documentation to indicate the use of appropriate reporting guidelines for health-related research (see [EQUATOR Network](http://www.equator-network.org/%20)), life science research (see the [BioSharing Information Resource](https://biosharing.org/)), or the [ARRIVE guidelines](http://www.plosbiology.org/article/info:doi/10.1371/journal.pbio.1000412) for reporting work involving animal research. Where applicable, authors should refer to any relevant reporting standards documents in this form.

If you have any questions, please consult our Journal Policies and/or contact us: [editorial@elifesciences.org](mailto:editorial@elifesciences.org).

**Sample-size estimation**

- You should state whether an appropriate sample size was computed when the study was being designed
- You should state the statistical method of sample size computation and any required assumptions
- If no explicit power analysis was used, you should describe how you decided what sample (replicate) size (number) to use

Please outline where this information can be found within the submission (e.g., sections or figure legends), or explain why this information doesn’t apply to your submission:

For the cell-signalling immunoblot analysis we typically show data for duplicate (Figure 1, 3A, 3S2, 5S1, 9S1C) or triplicate (Figure 3B, 3C, 3S1A-H, 5A, 5C, 6C, 9A, 9B, 9S1 A-B, 9S2, ) experiments and we repeat experiments at least twice, often more in order to ensure results are consistent (the number of repeats is stated in each figure legend). The main siRNA knock-down phosphatase screen described in Figure 2 was undertaken in triplicate and PPM1H emerged as the top hit in all 3 screens. We also include the primary data for each of the 3 siRNA screens (Fig 2 Figure Supplement 1, 2 and 3), in addition to the quantitation of the pRab10/Total Rab10 ratios in Supplementary Excel File 1. For CRISPR knock-out data in Figure 6A, we selected ten independent knock-out clones from three sets of guides and present the individual data for all of these to show variation between them and provide a representative result. For the time course and dose response experiments (data shown in Figure 7AB and Figure 7B) we show data for 8 separate time points or concentrations in duplicate for each condition to ensure reliability. For the immunolocalisation data in Figure 4 and 10, we show singlicate data, but at least 20 independent cells were analysed in each experiment and quantitation is provided in the main figure. For the phosphatase data in Figure 8 we show singlicate data, but similar results were obtained in 3 separate studies. We also used phosphatase inactive mutants to establish that catalytic activity observed was due to PPM1H phosphatase activity rather than a contaminating enzyme. For the ciliation in Figure 11 the average of duplicate determination is made at identical cell confluency; error bars represent SEM and the experiment was replicated 4 times by two lab members.

**Replicates**

- You should report how often each experiment was performed
- You should include a definition of biological versus technical replication
- The data obtained should be provided and sufficient information should be provided to indicate the number of independent biological and/or technical replicates
- If you encountered any outliers, you should describe how these were handled
- Criteria for exclusion/inclusion of data should be clearly stated
- High-throughput sequence data should be uploaded before submission, with a private link for reviewers provided (these are available from both GEO and ArrayExpress)

Please outline where this information can be found within the submission (e.g., sections or figure legends), or explain why this information doesn’t apply to your submission:

Information regarding experiment repeats and replicates can be found in the figure legends.

Data Availability: The mass spectrometry raw data and MaxQuant search output tables have been deposited to ProteomeXchange, PRIDE database (https://www.ebi.ac.uk/pride/archive/ unique identifier, PXD014794. Username: [reviewer47721@ebi.ac.uk](mailto:reviewer47721@ebi.ac.uk) Password: YUA9t9Dw)

All Plasmids, antibodies and proteins (including datasheets and sequence information) that we have generated for this study can be requested and information downloaded from MRC PPU Reagents and Services (https://mrcppureagents.dundee.ac.uk/).

**Statistical reporting**

- Statistical analysis methods should be described and justified
- Raw data should be presented in figures whenever informative to do so (typically when N per group is less than 10)
- For each experiment, you should identify the statistical tests used, exact values of N, definitions of center, methods of multiple test correction, and dispersion and precision measures (e.g., mean, median, SD, SEM, confidence intervals; and, for the major substantive results, a measure of effect size (e.g., Pearson's r, Cohen's d)
- Report exact p-values wherever possible alongside the summary statistics and 95% confidence intervals. These should be reported for all key questions and not only when the p-value is less than 0.05.

Please outline where this information can be found within the submission (e.g., sections or figure legends), or explain why this information doesn’t apply to your submission:

Precision measures used and p-values can be found in the figure legends.

(For large datasets, or papers with a very large number of statistical tests, you may upload a single table file with tests, Ns, etc., with reference to sections in the manuscript.)

**Group allocation**

- Indicate how samples were allocated into experimental groups (in the case of clinical studies, please specify allocation to treatment method); if randomization was used, please also state if restricted randomization was applied
- Indicate if masking was used during group allocation, data collection and/or data analysis

Please outline where this information can be found within the submission (e.g., sections or figure legends), or explain why this information doesn’t apply to your submission:

We feel that this does not apply for our article.

**Additional data files (“source data”)**

- We encourage you to upload relevant additional data files, such as numerical data that are represented as a graph in a figure, or as a summary table
- Where provided, these should be in the most useful format, and they can be uploaded as “Source data” files linked to a main figure or table
- Include model definition files including the full list of parameters used
- Include code used for data analysis (e.g., R, MatLab)
- Avoid stating that data files are “available upon request”

Please indicate the figures or tables for which source data files have been provided:

Supplementary Excel File 1 contains numerical data for graphs in Figure 2B and C.

Numerical data for Figure 8C can be found in Supplementary Excel File 2.

Data Availability: The mass spectrometry raw data and MaxQuant search output tables have been deposited to ProteomeXchange, PRIDE database (https://www.ebi.ac.uk/pride/archive/ unique identifier, PXD014794. Username: [reviewer47721@ebi.ac.uk](mailto:reviewer47721@ebi.ac.uk) Password: YUA9t9Dw)

All Plasmids, antibodies and proteins (including datasheets and sequence information) that we have generated for this study can be requested and information downloaded from MRC PPU Reagents and Services (https://mrcppureagents.dundee.ac.uk/).
